# Wearable Camera-Based Dietary Assessment of Mother–Father Dyads in Urban and Rural Households in Ghana

**DOI:** 10.1016/j.cdnut.2026.107696

**Published:** 2026-04-18

**Authors:** Christabel A Domfe, Megan A McCrory, Tom Baranowski, Edward Sazonov, Tonmoy Ghosh, Viprav Raju, Gary Frost, Matilda Steiner-Asiedu, Mingui Sun, Wenyan Jia, Benny Lo, Alex K Anderson

**Affiliations:** 1 Department of Nutritional Sciences, University of Georgia, Athens, GA, United States; 2 Department of Health Sciences, Boston University, Boston, MA, United States; 3 USDA/ARS Children's Nutrition Research Center, Department of Pediatrics, Baylor College of Medicine, Houston, TX, United States; 4 Department of Electrical and Computer Engineering, University of Alabama, Tuscaloosa, AL, United States; 5 Section for Nutrition Research, Department of Metabolism, Digestion and Reproduction, Imperial College, London, United Kingdom; 6 Department of Nutrition and Food Science, University of Ghana, Accra, Ghana; 7 Department of Electrical and Computer Engineering, University of Pittsburgh, Pittsburgh, PA, United States; 8 Hamlyn Center, Imperial College, London, United Kingdom

**Keywords:** mother–father dyads, urban/rural households, energy and nutrient intakes, dietary assessment, Automatic Ingestion Monitor-2, Ghana

## Abstract

**Background:**

There is a dearth of studies examining the dietary intakes of parents from low- and middle-income countries, which are foundational for understanding the household-level double burden of malnutrition (DBM).

**Objectives:**

To assess the energy and nutrient intakes (carbohydrates, protein, fat, vitamin A, folate, zinc, and iron) of mother–father dyads from rural and urban households in Ghana using a wearable camera, an objective measure of dietary intake, not subject to self-reporting errors.

**Methods:**

In this cross-sectional study, purposive convenience sampling was used to recruit 60 households with a mother, father, and a child younger than 5 y and/or an adolescent from 1 rural and 1 urban community in Ghana. Both parents wore the Automatic Ingestion Monitor 2 (AIM-2) over 2 weekdays and 1 weekend day. Dietary intake was analyzed using custom AIM Annotation Software. Household characteristics were collected via questionnaire, and body mass index (kg/m^2^) was calculated from measured height and weight.

**Results:**

No differences in mean energy, macronutrient, or micronutrient intakes were observed between mothers and fathers within either area of residence. Dyad-level analyses showed that in rural households, mothers had higher caloric (*P* = 0.001), vitamin A (*P* = 0.048), and zinc intakes (*P* = 0.001) than fathers, who had higher iron intakes (*P* < 0.001) than mothers. In urban households, mothers had higher caloric (*P* = 0.006) and zinc intakes (*P* < 0.001) than fathers who had higher iron intakes (*P* < 0.001) than mothers. The prevalence of overweight/obesity was higher among mothers than fathers in both urban (70% versus 40%, *P* = 0.020) and rural (67% versus 30%, *P* = 0.005) households.

**Conclusions:**

The coexistence of overweight/obesity and low iron intakes among mothers highlights a unique presentation of the DBM at the household level. The AIM-2 shows promise for addressing limitations of traditional dietary assessment methods through passive and objective image capture.

## Introduction

The manifestation of the double burden of malnutrition (DBM), characterized by the coexistence of undernutrition and overnutrition (overweight and obesity), has changed over the years, shifting from higher prevalence in higher-income low- and middle-income countries (LMICs) to now higher rates in poorer LMICs [1]. Studies examining the DBM have primarily focused on mother–child dyads [[2], [3], [4], [5], [6]], without much attention given to other household members. Even more limited in the literature are studies that have explored the caloric and nutrient intakes of parents, which are foundational to examining the prevalence of DBM at the household level. Examining the dietary intake of both mothers and fathers is essential for understanding household food-sharing dynamics [7] and gender roles [8], which can influence how nutrition interventions are received and practiced within households. These dynamics may either reinforce or challenge efforts to address the DBM within households in LMICs.

Within LMICs, nutritional challenges have often differed between urban and rural settings. However, these are changing, with rural communities increasingly experiencing nutrition-related exposures traditionally associated with urbanization [9]. These include changes in the food environment [10], the consumption of ultraprocessed foods as socioeconomic status improves [[11], [12], [13]], a decline in fruit and vegetable consumption [14], and declining rates of physical activity [15], resulting in a rising prevalence of nutrition-related noncommunicable diseases [16,17]. In Ghana, an LMIC in the sub-Saharan region of Africa [18], the nutrition transition is hugely implicated in the changing trends [19]. The availability and consumption of highly processed foods are no longer exclusive to high-income countries and urban settings.

To implement effective programs and policies to address the DBM, the assessment of food and nutrient intake must be reliable and accurate. However, because of reliance on interviewer-assisted paper-based questionnaires, dietary assessment in LMIC settings is considered costly, time consuming, and burdensome for both the respondent and interviewer [[20], [21], [22], [23]]. This, together with the challenge of portion size estimation inaccuracies [21] and the dearth of country-representative food composition databases, compounds the challenges of dietary assessment in LMICs [21,23,24]. Wearable sensor technologies capture food consumption and enable the estimation of food intake objectively and passively, that is, without the user actively recording or reporting intake [25], making them especially suitable for settings with low literacy levels [20]. Their potential use in LMICs is promising, largely due to the reduced user burden and bias, as well as the ability to provide detailed, real-time dietary intake data.

In this study, the intakes of energy, macronutrients (carbohydrate, protein, and fat), and micronutrients of public health concern (iron, zinc, folate, and vitamin A) [26] of mother–father dyads were assessed, examining whether there were differences by urban residence or rural residence. We hypothesized that fathers would have higher energy and nutrient intakes than mothers due to greater physiological needs and that this difference would be particularly evident among men in urban households, where patriarchal cultural norms influence food sharing and consumption patterns to favor males.

## Methods

Data for the present study come from a project that was carried out in 3 phases, as described previously [27]:

Phase 1/study 1: The formative study involved conducting key informant interviews with selected household members [28].

Phase 2/study 2: The feasibility study evaluated the acceptability and usability of the 3 imaging devices: the AIM-2 (camera + sensor affixed to eyeglasses), the eButton (camera pinned to the shirt/blouse or worn around the neck of participants), and the FoodCam (camera device mounted over the food preparation and food consumption areas) in Ghana [27].

Finally, phase 3/study 3: The field validation study tested the accuracy of the 3 imaging devices against the weighed food records and image-assisted 24-h recalls. The data used in this present study come from the field validation study. A cross-sectional community-based design was used. Data were collected on 2 weekdays and 1 weekend day in households from both rural and urban communities in Ghana. Data collection started in the rural community from November 2020 to February 2021 and took place in the urban community from March to May 2021. The study was approved by the Human Subjects Institutional Review Boards of the University of Georgia (STUDY00006121) and the Noguchi Memorial Institute of Medical Research, University of Ghana (#-046/18-19). Written informed consent was obtained from all participants.

### Ghana

Ghana, an LMIC in the sub-Saharan African region [29], is bordered by the Atlantic Ocean to the south, Togo to the east, Burkina Faso to the north, and Côte d’Ivoire to the west. The current population stands at about 32 million, with 59% residing in urban areas and 41% residing in rural areas [29]. As at 2023, there were 2.1 million undernourished Ghanaians [30]. Almost 13.3% of the adult population was reported to have obesity in 2023 [31]. The country is facing the DBM, which has a significant impact on the health and gross productivity of the nation [32].

In the Greater Accra Region, the University of Ghana Junior Staff Residence was chosen as the urban study site, whereas a rural community in the Eastern Region, *Asaase Kokoo* (which literally translates as “a land of red soil”), was the rural study site. The University of Ghana Junior Staff Residence is located across the main University of Ghana campus, on the northern side of the campus. It was established to provide housing for junior staff members, such as administrative assistants, artisans, cooks, laboratory assistants, and drivers. *Asaase Kokoo* is a rural settlement in the Akuapem North Municipal Assembly in the southeast part of the Eastern Region of Ghana. Most of the residents engage in agricultural production—cultivating food crops, rearing livestock, and brewing local alcoholic beverages on farms.

The principal investigator, together with the field coordinator, led the community entry, a means of initiating and cultivating relationships with community leaders and residents, at both study sites before the start of the study. At *Asaase Kokoo,* the chief of the town and community elders were informed, and their permission was obtained to begin the study. The *mmerantehene* (leader of the youth) helped with the identification and recruitment of eligible households. At the University Junior Staff Residence, the assemblyman—the elected leader of the community at the municipal level—was informed, permission was sought, and then approval was obtained to conduct the study. A community member was identified who helped the field research team in the recruitment of eligible households in this area. Purposive convenience sampling was used for household recruitment. An eligible household was defined as one in which both parents (mother and father) lived together with a child younger than 5 y and/or an adolescent. Another inclusion criterion was that all eligible household members had to partake in ≥1 household eating occasion each day. Sixty households participated, 30 each from the urban and rural communities.

In the present study, only data collected with the AIM-2 are reported, as it was the only device worn by both mothers and fathers within the households. The AIM-2 was used to assess the food intake of household members in their natural environments, without any restrictions or interference from the research team. The day before data collection started in an identified household, research staff took the household through the consent process and obtained consent and/or assent (for children) from the household members who were eligible for participation. The field research staff then described the study protocol to the eligible household members, trained the participants on how and when to wear the device, and answered any questions they had about participation.

### Data collection

Two trained bilingual research assistants were assigned to each household for a 3-d data collection period, which included 2 weekdays and 1 weekend day to capture habitual dietary intake using the AIM-2. On each data collection day, the assistants arrived around the time household members woke up (∼006:30) and stayed until all food-related activities concluded (around 18:00–19:00 in the rural community and 20:00 in the urban community). They remained in the vicinity of the household throughout the day, remaining unobtrusive, stepping in only to ensure that devices were correctly mounted or worn, and taking weighed food measurements. Findings from the larger validation study, which compares the gram-level and nutrient intake estimates from the wearable cameras and weighed food records, are reported in a different article [33]. Mothers and fathers were asked to wear the AIM-2, affixed to eyeglasses, from wake time to bedtime. Participants were not restricted but encouraged to go about their usual daily routines during the days of data collection, including activities outside the home, such as work, church, funerals, grocery shopping, etc. They were advised to remove the device during private activities (e.g., using the bathroom).

In addition to wearing the AIM-2, the research assistants administered a household sociodemographic questionnaire and took anthropometric measurements using an SECA 769 Physician scale. Weight was recorded to the nearest 0.1 kg, and height was measured to the nearest 0.1 cm to compute BMI (in kg/m^2^). At the end of the 3 d, each household received compensation of GH¢600.00 (equivalent to $100 at the time) in appreciation for their participation.

### The AIM technology (device and software)

The AIM technology comprises the AIM-2 wearable camera and AIM Annotation Software [34]. The AIM-2 is a wearable sensor technology for objectively assessing food intake [27,35,36] passively, that is, with no active input or actions needed from the user other than to wear the device. The USB port, camera lens, and sensor are its 3 main components. The user wears the AIM-2 attached to eyeglasses. Data download and device charging are made possible via the USB port. For this study, the AIM-2 was programmed to automatically capture images every 15 s of the area in front of the wearer, including food preparation and consumption throughout the entire time it was worn. The sensor and an internal accelerometer work to detect food and beverage consumption. The captured images and sensor data were downloaded from the device and uploaded to a secure, password-protected cloud data storage at the end of each data collection day by the field coordinator. The device was then recharged overnight for data collection the following day. An engineering team accessed the uploaded data and processed the images into a format readable by the AIM Annotation Software [34].

The interface of AIM Annotation Software, version 4.5 includes the image panel, sensor panel, and meal data panel. The image panel allows the nutritionist to review the full sequence of food consumption, including the start and end of eating episodes, the types of foods consumed, and food-sharing behavior. Each image is time-stamped, helping classify eating occasions as breakfast, lunch, dinner, or snacks. The sensor panel displays sensor peaks that typically align with eating activity, helping validate the timing and duration of consumption. The meal data panel presents the estimated dietary intake values based on annotated portion sizes.

Portion size estimation was performed by the nutritionist using the sequential images from the image panel. Both the quantity of food consumed and any leftovers were visually estimated using a photographic food atlas for reference [37]. Before performing the image annotation and portion size estimation, the nutritionist was trained with food images of known weights and portion sizes [38,39] using data from the phase 2 feasibility study, which evaluated the acceptability and usability of the AIM-2 device. This included hundreds of food images and weighed food record data. After this training, the nutritionist completed a test with 20 images requiring correct food identification and portion estimation within a mean of ±15% of actual weights. For any images that were overestimated or underestimated, additional images were provided for a second round of testing, after which the nutritionist met the predetermined accuracy criteria. The nutritionist was deemed trained after the food identification was 100% accurate, and the agreement between the estimated and actual portion sizes was within a mean of ±15% of actual weights.

Portion size was estimated by estimating the amount of food in the image just before eating began and again in the last image showing leftover food, then calculating the difference. The corresponding food weights from the food atlas were used to estimate portion sizes. Then, an appropriate database item was chosen, and the software automatically calculated energy and nutrients consumed, that is, the macronutrients (carbohydrate, protein, and fat), fiber, and micronutrients. For mixed dishes, the components were first identified, proportions were then estimated, and these proportions were converted to gram weights using corresponding values from the photographic food atlas. In rare situations where a food item was not found in any of the 5 databases, the closest match was selected and used after consulting with and obtaining approval from the senior nutritionist who oversaw the annotation process. Of interest to this study were the contents of energy, macronutrients, and selected micronutrients of public health importance in Ghana (vitamin A, folate, zinc, and iron).

### Energy and nutrient intake analysis

Once the data were uploaded into the AIM Annotation Software, the processed images were made visible within the software interface. To observe the eating dynamics fully and not miss a food consumption episode, processed images that were uploaded into the software were individually reviewed by the nutritionist. The food composition databases programmed into the annotation software were used in the following order of priority: West African [40], Food and Nutrient Database for Dietary Studies [41], Kenyan [42], Ugandan [43], and the United States Department of Agriculture [44] food composition databases for nutrient intake analysis [34]. The closest match of food consumed was selected by the nutritionist during the image annotation process from one of the databases if the exact food was not identified in the West African database.

### Statistical analysis

The daily energy and nutrient intake output for each participant-day was provided by the AIM Annotation Software in Microsoft Excel files. The data of interest were manually compiled into a single spreadsheet before being imported to SPSS (IBM SPSS, version 27) and SAS (SAS Institute Inc.) Studio v9.4 for statistical analysis. Descriptive statistics were calculated and reported as frequencies and percentages (categorical variables) and means ± SE (continuous variables) by performing Fisher’s exact test for BMI categories and sociodemographic variables among urban and rural households. BMI categories were dichotomized as underweight/normal (≤24.9) and overweight/obese (>24.9). The 3-d means of energy and nutrient intake were calculated, and comparisons were made between urban and rural households. Mother–father dyad differences in caloric intake were calculated by first dividing each parent’s 3-d average caloric intake by their respective recommended daily caloric intakes based on Ghana’s Ministry of Health guidelines (i.e., 2100 kcal/d for a moderately active woman and 2700 kcal/d for a moderately active man) [45]. The resulting percentages were then used to compute the dyad difference by subtracting the father’s percentage from the mother’s percentage. The percentage contribution of each macronutrient to total daily calories was calculated separately for mothers and fathers by first converting mean macronutrients to calories (multiplying by 4 kcal for carbohydrates and proteins and 9 kcal for fats) and then dividing by the total calories. Mother–father dyad differences in micronutrient intakes were assessed by calculating individual intakes separately for mothers and fathers and expressing intake as a percentage of their respective Institute of Medicine requirements [46]—that is, 700 versus 900 μg retinol activity equivalents for vitamin A, 400 μg dietary folate equivalents for folate, 8 versus 11 mg for zinc, and 18 versus 8 mg for iron for women and men, respectively. Dyad difference variables were then created by subtracting the fathers’ value from the mothers’ value for each nutrient.

Descriptive statistics were computed for these dyad differences. A 1-sample *t* test was used to determine whether the mean difference significantly differed from 0, within each location, whereas an unpaired *t* test was conducted to compare the dyad differences across locations. Differences in mothers’ and fathers’ mean caloric and nutrient intakes by BMI category were assessed using an unpaired *t* test, stratified by household location (urban versus rural). Statistical and marginal significances were determined at *P* ≤ 0.05 and *P* < 0.10, respectively, for all analyses.

## Results

Household characteristics are described previously in the study by Domfe et al. [47]. Briefly, the mean age ± SD of fathers in rural households was 39.6 ± 12.4 y compared with 44.9 ± 8.7 y among urban fathers (*P* = 0.008). For mothers, the mean age in rural households was 34.5 ± 10.7 y, whereas urban mothers had a mean age of 44.4 ± 7.4 y (*P* < 0.001). Close to half of rural households (47%) had a household size of ≤4, compared with about 87% of urban households that had a household size of ≥5 (*P* = 0.014). Additionally, 60% of rural parents owned their homes compared with 3% of urban parents (*P* = 0.001). Within each location, BMI category distribution differed between mothers and fathers (*P* = 0.020 for urban, *P* = 0.005 for rural) (Table 1). Overweight/obesity was more prevalent among mothers than fathers, regardless of location, with 70% of mothers compared with 40% of fathers in urban households and 67% compared with 30% of fathers in rural households having overweight/obesity.

**TABLE 1:** Comparison of BMI distributions of mothers and fathers within and across rural and urban households in Ghana

Weight status	Urban *n* (%)		*P* value^1^	Rural *n* (%)		*P* value^1^	Urban vs. rural *P* values^2^	
	Mothers	Fathers	0.020	Mothers	Fathers	0.005	Mothers	Fathers
≤24.9 kg/m^2^	9 (30)	18 (60)		10 (33)	21 (70)		0.781	0.417
>24.9 kg/m^2^	21 (70)	12 (40)		20 (67)	9 (30)			

Individuals with underweight/normal weight: ≤24.9 kg/m^2^; individuals with overweight/obese: >24.9 kg/m^2^.

1 Within location comparison of BMI distributions.^2^Across urban and rural household comparison of BMI distributions for mothers and fathers.

### Energy and nutrient intake

Over 5,500,000 images were captured by all imaging devices used in the study across urban and rural participants over the 3-d data collection period. There were no differences in the mean intakes of energy, macronutrients, and micronutrients for mothers and fathers within urban and rural households (Table 2). Across locations, urban mothers had higher intakes of fat (*P* = 0.007), vitamin A (*P* = 0.005), and folate (*P* = 0.022) than rural mothers. Urban fathers had higher fat intake (*P* = 0.019) than rural fathers, who had higher iron intakes (*P* = 0.024) than urban fathers.

**TABLE 2:** Comparison of mothers’ and fathers’ mean energy and nutrient intakes within and across urban and rural households in Ghana using the Automatic Ingestion Monitor-2

Variable	Urban			Rural			Urban vs. Rural *P* value^2^	
	Mothers mean ± SE *N* = 30	Fathers mean ± SE *N* = 30	*P* value^1^	Mothers mean ± SE *N* = 30	Fathers mean ± SE *N* = 30	*P* value^1^	Mothers	Fathers
Energy (kcal)	1691 ± 95	1648 ± 109	0.766	1596 ± 83	1733 ± 105	0.308	0.450	0.577
CHO (g)	242 ± 13	226 ± 16	0.457	253 ± 13	276 ± 17	0.285	0.564	0.038
Protein (g)	50.6 ± 3.1	54.5 ± 4.1	0.445	49.7 ± 3.1	54.5 ± 3.3	0.295	0.843	0.997
Fat (g)	52.1 ± 4.8	54.8 ± 4.7	0.691	36.9 ± 2.5	41.2 ± 3.0	0.278	0.007	0.019
Vitamin A (μg RAE)	1151.8 ± 227.0	898.8 ± 161.0	0.367	467.8 ± 65.3	541.2 ± 97.5	0.534	0.005	0.062^3^
Folate (μg DFE)	254.1 ± 30.4	221.0 ± 19.7	0.365	176.9 ± 12.3	199.0 ± 15.4	0.266	0.022	0.382
Zinc (mg)	7.7 ± 0.5	7.9 ± 0.6	0.805	8.0 ± 0.5	8.7 ± 0.6	0.361	0.659	0.340
Iron (mg)	17.7 ± 1.1	17.9 ± 1.3	0.924	21.4 ± 1.7	23.7 ± 2.2	0.403	0.076	0.024

Values are 3-day mean ± SE.

CHO, carbohydrates; DFE, dietary folate equivalent; RAE, retinal activity equivalent.

1 Significant main effect of mothers and fathers within a location.

2 Significant main effects of mothers and fathers across a location.

3 Marginal significance at *P* < 0.10.

Dyad difference analysis within urban households showed mothers having higher energy (*P* = 0.001), vitamin A (*P* = 0.048), and zinc (0.001) intakes relative to requirements than fathers, whereas fathers had higher iron intakes (*P* < 0.001) relative to requirements than mothers (Table 3). Within rural households, mothers had higher energy (*P* = 0.006) and zinc (*P* < 0.001) intakes, whereas fathers had higher iron (*P* < 0.001) intakes than mothers relative to requirements. Dyad differences across urban and rural households were not different from each other.

**TABLE 3:** Mother–father dyad differences in energy and nutrient intakes within and across urban and rural households in Ghana

Nutrient	Mean urban dyad difference^1^ ± SE (% of requirement) *N* = 30	*P* value	Mean rural dyad difference^1^ ± SE (% of requirement) *N* = 30	*P* value	Urban vs. rural *P* value
Energy^2^	19.5 ± 5.4	0.001	11.8 ± 4.0	0.006	0.253
Carbohydrate^3^	2.7 ± 1.8	0.136	−0.1 ± 0.9	0.945	0.170
Protein^3^	−1.3 ± 0.8	0.091^5^	−0.3 ± 0.2	0.118	0.194
Total fat^3^	−2.1 ± 1.9	0.279	−0.6 ± 1.1	0.583	0.493
Vitamin A^4^	64.7 ± 31.3	0.048	6.7 ± 6.4	0.302	0.079^5^
Folate^4^	8.3 ± 9.6	0.398	−5.5 ± 3.9	0.164	0.192
Zinc^4^	24.3 ± 6.8	0.001	20.7 ± 5.4	<0.001	0.679
Iron^4^	−125.0 ± 15.9	<0.001	−177.6 ± 22.0	<0.001	0.058

1 Dyad difference = %intake of mothers − %intake of fathers.

2 Mean dyad difference for calories was calculated from the differences in the percentages of the respective recommended daily intakes.

3 Mean dyad differences for the macronutrient are calculated from differences in intakes relative to the % total energy.

4 Mean dyad differences for the micronutrients are calculated from the mean differences relative to requirements.

5 Indicates marginal significance at *P* < 0.10.

Within urban households, mean energy and nutrient intakes did not differ by BMI category (Supplemental Table 1). In contrast, within rural households, mothers with overweight/obesity had lower vitamin A intakes than mothers with normal weight (*P* = 0.037), whereas fathers with overweight/obesity had marginally higher fat (*P* = 0.057) and vitamin A (*P* = 0.061) intakes than fathers with normal weight (Supplemental Table 2).

## Discussion

In this study, the AIM-2, a wearable camera mounted on eyeglasses that passively captures images of dietary intake, was used to objectively assess the energy and nutrient intakes of mother–father dyads in rural and urban households in Ghana. In our a priori hypothesis, we expected higher energy and nutrient intakes for fathers, especially those in urban households. However, the results showed deviations from this expectation—the mean caloric and nutrient intakes did not differ between mothers and fathers within either urban or rural households. Additionally, dyad comparison relative to respective requirements within urban households showed that mothers had higher caloric, vitamin A, and zinc intakes compared with fathers. In contrast, fathers had higher iron intakes compared with mothers. Similarly, within rural households, mothers had higher caloric and zinc intakes compared with fathers, who had higher iron intakes than mothers. Although mothers with overweight and obesity were most common, irrespective of location, their lower iron intakes relative to requirements compared with fathers highlight a unique presentation of the DBM at the household level. Within both urban and rural households, fathers were generally older than mothers. This pattern is consistent with previous studies documenting older ages among husbands in patriarchal settings [48] and in low-socioeconomic contexts [49] compared with wives. Prior research has also shown wider spousal age gaps among individuals with lower versus higher educational attainment [50], whereas similar to our study, urban residents have been associated with smaller spousal age gaps [51].

The mean caloric intakes for the dyads, irrespective of area of residence, fell below general recommendations [46]. Poverty levels remain a major driver, with 18% of the population living in extreme poverty in 2023 [30] and significantly higher poverty rates in rural areas compared with urban regions [52]. Among the lowest income deciles in Sub-Saharan Africa, including Ghana, caloric consumption often falls below recommendations [53]. Given the data on poverty levels, it is unsurprising that both urban and rural dyads failed to meet recommended calorie intakes, highlighting the role of financial challenges in limiting the ability to meet adequate nutritional needs [[54], [55], [56]] and the critical need for targeted interventions in LMICs. Also, participants in this study may have consumed less than usual due to social desirability bias associated with the presence of field staff around the home. In addition, some meals consumed outside the home may not have been captured, particularly among urban fathers, where the absence of field staff may have reduced compliance with device wearing, although the study protocol required participants to continue wearing the AIM-2 even when outside the home.

We sought to explore whether there were differences in the mean intakes of calories and nutrients by urban or rural residents in Ghana [57,58]. Our findings indicated differences in fat and vitamin A intakes by location, with both urban mothers and fathers consuming more than their rural counterparts. Urban mothers also had higher folate intakes compared with rural mothers, whereas rural fathers had higher iron intakes than urban fathers. Despite these differences, comparisons of dyad intake of energy and nutrients across locations revealed no differences. A previous study among Ghanaian adults identified similar dietary trends among rural and urban households, highlighting the widespread availability of shops/stalls selling highly processed snacks and beverages in rural areas as a driver of this trend [59]. It is worth highlighting that although cooking methods and recipes used within households differed across both areas of residence, the use of standardized recipes from the AIM-2 Annotation software’s food composition databases may have minimized these differences to an extent. Regardless, our findings support previous research that highlights changing dietary patterns in rural households to closely resemble urban household patterns, consistent with the nutrition transition [9].

This study highlights a unique depiction of the DBM, with mothers showing higher BMI yet lower iron intakes, irrespective of location. Exploration of caloric and nutrient intake by BMI categories showed no statistically significant differences in intakes for mothers and fathers with normal weight compared with overweight/obese within urban households. On the contrary, mothers with overweight/obesity had lower vitamin A intakes than mothers with normal weight within rural households, and rural fathers with overweight/obesity had marginally higher fat intakes than fathers with normal weight. Although this study did not assess any biochemical measures, our findings are consistent with the trend of overweight and anemia being the most common presentation of the DBM among Ghanaian women [60]. Thus, we provide dietary evidence through an objective method, supporting previous findings. Cultural factors also underpin the overweight/obesity patterns observed among the mothers. In many African societies, men traditionally encourage their wives to increase food intake to achieve a plump physique, signaling to in-laws that their daughters are well cared for in marriage [61]. This cultural practice contributes to the disproportionate prevalence of overweight and obesity among adult women in African societies, where the nutrition transition alone does not fully explain these trends [62]. Seasonality as a determinant of micronutrient status has been well established in previous studies [63]. Data collection occurred in the rural community from November 2020 to February 2021 (dry season) and in the urban community from March to May 2021, coinciding with the mango fruiting season. This seasonal difference may explain the higher mean vitamin A intakes in urban households, particularly among mothers. That notwithstanding, vitamin A deficiency is considered a contributor to the prevalence of anemia [64], whereas folate deficiency also manifests as megaloblastic anemia. Together with iron deficiency anemia, our dietary intake results indicate a need for tailored dietary interventions to improve the micronutrient status of women, irrespective of area of residence.

In this study, we demonstrated the utility of an objective dietary assessment tool to investigate the energy and nutrient intakes of mother–father dyads living together in urban and rural Ghanaian households. The AIM-2 captured dietary intake data passively, requiring only that participants wear eyeglasses with the device attached. This approach potentially reduces recall bias associated with 24-h recalls and lowers participant burden, thereby likely minimizing social desirability bias and overcoming key limitations of the weighed food record, the gold standard for dietary assessment [33,38]. Importantly, this method could potentially reduce the logistical and training costs associated with the commonly used paper-and-pen method in LMICs. Because of the passive nature of data collection, it does not rely on the numeracy skills of the participants for portion size estimation. This study helps address an important research gap on dietary intake among parents in LMICs [61]. To the best of our knowledge, this is the first study to conduct such an investigation. A strength of this study is that a single nutritionist performed all portion size estimations, ensuring consistency.

A limitation of this study is the lack of a Ghanaian-specific food database with recipes representing both urban and rural areas in the custom AIM Annotation Software. Additionally, we did not collect data related to physical activity levels of the participants. Instead, we applied conservative energy requirements for the caloric intake comparisons between mothers and fathers, which may not accurately reflect the true energy needs of the participants. The study findings, specifically regarding energy and nutrient intakes by BMI categories, should be interpreted as exploratory because of the increased risk of type 1 error associated with conducting multiple statistical tests. Finally, with urban fathers spending most of their time away from home, noncompliance with device wearing may have resulted in not capturing all the meals that were consumed.

In conclusion, the energy and nutrient intakes of mother–father dyads across urban and rural households in Ghana were similar, with a presentation of the DBM at the household level. Our technological approach to dietary assessment addresses challenges associated with traditional methods by simplifying data collection and enabling portion size estimation through image capture and facilitating dietary data analysis without relying on the participants’ memories. The AIM-2 showed promise in overcoming many of the challenges of traditional dietary assessment methods in an LMIC grappling with the DBM.

## Author contributions

The authors’ responsibilities were as follows – CAD: performed image annotation, nutritional and statistical analysis, and drafted the manuscript; MAM and AKA: established procedures for and oversaw image annotation. MAM, TB, and AKA: revised the initial draft manuscript; VR, TG, and ES: processed the AIM-2 images for the AIM Annotation Software; MAM, AKA, ES, WJ, BL, GF, MSA, TB, and MS: were involved in the concept development and review of the draft manuscript; AKA and MS: oversaw the project implementation, coordination, and data collection in Ghana; and all authors reviewed and approved the manuscript.

## Data availability

Data described in the manuscript, code book, and analytic code will be made available upon request, either before or after publication.

## Declaration of generative AI and AI-assisted technologies in the writing process

The author(s) declare that no generative AI or AI-assisted technologies were used in the writing of this manuscript.

## Funding

This work was supported by the Bill & Melinda Gates Foundation (Contract INV: 006713). The funder was not involved in the study design, data collection, analysis, interpretation, manuscript writing, or submission for publication.

## Conflict of interest

The authors report no conflicts of interest.
