# Supplementary material for: Wearable Camera-Based Dietary Assessment of Mother–Father Dyads in Urban and Rural Households in Ghana
Source: Curr Dev Nutr. 2026 Apr 18;10(5):107696. doi: 10.1016/j.cdnut.2026.107696 (PMC13194622; doi:10.1016/j.cdnut.2026.107696)
Supplement: Multimedia component 1 [file mmc1.docx]

**Supplemental Table 1: Mean energy and nutrient intakes by BMI categories within urban households in Ghana**

| **Variable** | **Urban** | | | | | |
| --- | --- | --- | --- | --- | --- | --- |
|  | **Mothers** | | | **Fathers** | | |
|  | Underweight/normal; n=9 | Overweight  /Obese; n=21 | p-value^1^ | Underweight/normal; n=18 | Overweight/ obese; n=12 | p-value^1^ |
| Energy (kcal) | 1589.0±98.0 | 1735.4±129.2 | 0.374 | 1686±133.3 | 1591.5±192.4 | 0.679 |
| CHO (g) | 234.4±21.2 | 245.3±17.0 | 0.714 | 239.3±21.1 | 207.2±24.5 | 0.335 |
| Protein (g) | 47.2±4.7 | 52.0±3.9 | 0.476 | 54.9±4.3 | 53.9±8.3 | 0.909 |
| Fat (g) | 50.2±5.8 | 53.0±6.5 | 0.798 | 52.8±5.3 | 58.0±9.1 | 0.599 |
| Vit. A (µg RAE) | 1118.6±376.3 | 1165.9±287.1 | 0.926 | 847.4±181.2 | 975.7±306.3 | 0.703 |
| Folate (µg DFE) | 237.7±42.0 | 261.1±40.1 | 0.730 | 212.8±20.5 | 233.3±39.6 | 0.618 |
| Zinc (mg) | 6.6±0.6 | 8.1±0.7 | 0.177 | 8.4±0.7 | 7.1±1.2 | 0.355 |
| Iron (mg) | 15.8±4.7 | 18.5±6.3 | 0.252 | 18.6±1.7 | 16.7±2.2 | 0.489 |

CHO: Carbohydrates; Values are 3-day mean ± SE.

^1^ Significant main effect of normal vs overweight/obese parent **within** a location

Mothers: n(underweight) = 0; n(normal) = 9; n(overweight)=10; n(obese) = 11

Fathers: n(underweight) = 1; n(normal) = 17; n(overweight)=10; n(obese) = 2

**Supplemental Table 2: Mean energy and nutrient intakes by BMI categories within rural households in Ghana**

| **Variable** | **Rural** | | | | | |
| --- | --- | --- | --- | --- | --- | --- |
|  | **Mothers** | | | **Fathers** | | |
|  | Normal; n=10 | Overweight/ Obese; n=20 | p-value^1^ | Normal; n=21 | Overweight/ obese; n=9 | p-value^1^ |
| Energy (kcal) | 1482.7±130.5 | 1652.2±105.4 | 0.343 | 1638±110.0 | 1953.6±231.6 | 0.173 |
| CHO (g) | 238.2±22.6 | 260.1±15.9 | 0.434 | 262.9±18.6 | 305.5±35.3 | 0.253 |
| Protein (g) | 44.7±5.2 | 52.3±3.8 | 0.250 | 52.8±3.7 | 58.4±7.2 | 0.451 |
| Fat (g) | 33.7±3.9 | 38.6±3.3 | 0.378 | 37.5±3.0 | 50.0±6.7 | **0.057*** |
| Vit. A (µg RAE) | 657.9±148.9 | 372.7±55.6 | **0.037** | 422.3±83.7 | 818.6±245.7 | **0.061*** |
| Folate (µg DFE) | 187.9±24.1 | 171.4±14.2 | 0.535 | 190.3±20.0 | 219.3±21.1 | 0.397 |
| Zinc (mg) | 7.7±0.8 | 8.1±0.6 | 0.659 | 8.2±0.7 | 9.8±1.2 | 0.242 |
| Iron (mg) | 18.3±1.9 | 22.9±2.4 | 0.215 | 22.0±2.2 | 27.7±5.0 | 0.225 |

CHO: Carbohydrates; Values are 3-day mean ± SE.

^1^ Significant main effect of normal vs overweight/obese parent **within** a location

*Indicates marginal significance at p<0.10

Mothers: n(underweight) = 0; n(normal) = 10; n(overweight)=10; n(obese) = 10

Fathers: n(underweight) = 1; n(normal) = 20; n(overweight)=7; n(obese) = 2
